# Site-Specific and Quantitative O‑GlcNAc Proteomics for Hepatocellular Carcinoma

**DOI:** 10.1021/acs.jproteome.5c00939

**Published:** 2026-03-11

**Authors:** Chunyan Hou, Ping Li, Ethan Pei, Hemeng Zhang, Ci Wu, Jingtao Deng, Stephen W. Byers, Junfeng Ma

**Affiliations:** † Department of Oncology, 8368 Lombardi Comprehensive Cancer Center, Georgetown University Medical Center, Washington D.C. 20007, United States; ‡ Department of Oncology, Renmin Hospital of Wuhan University, Wuhan 430064, China

**Keywords:** O-GlcNAc, O-GlcNAcylation, proteomics, HCD-pd-EThcD, liver cancer

## Abstract

O-linked β-*N*-acetylglucosamine (O-GlcNAc) modification (O-GlcNAcylation) underlies the pathogenesis of multiple cancers, including hepatocellular carcinoma (HCC). However, comprehensive and quantitative characterization of site-specific O-GlcNAcylation at the proteome scale remains technically challenging. Here, we employed an integrated workflow for the quantitative O-GlcNAc proteomics of HCC and controls. Proteins from liver samples were subjected to chemoenzymatic labeling, photocleavable alkyne-biotin-based enrichment, proteolytic digestion, and isotopic labeling with tandem mass tags. The O-GlcNAc peptides were analyzed by a nanoUPLC-MS/MS system in HCD product-dependent EThcD (HCD-pd-EThcD) mode for site mapping and quantification. A total of 440 O-GlcNAc peptides, representing 305 sites on 196 proteins, were confidently identified. Differential analysis revealed 190 O-GlcNAc peptides from 121 proteins significantly upregulated in HCC after normalization to their corresponding protein abundance. Functional enrichment and protein–protein interaction analyses indicate that proteins with increased levels of O-GlcNAcylation are involved in nuclear transport, transcriptional regulation, and ATP-dependent chromatin remodeling. Our work provides quantitative proteomic insights into O-GlcNAcylation in HCC, revealing global upregulation and functional clustering of O-GlcNAc-modified proteins. These findings will help elucidate the functional roles of O-GlcNAcylation in liver cancer, facilitating the development of novel therapeutics and sensitive biomarkers.

## Graphical abstract

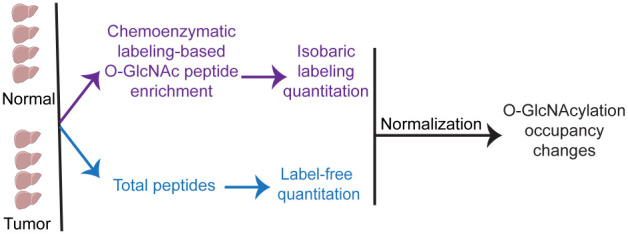

## Introduction

O-linked β-*N*-acetylglucosamine (O-GlcNAc) modification, a reversible post-translational modification (PTM), involves the attachment of a single *N*-acetylglucosamine (GlcNAc) moiety to the hydroxyl groups of serine, threonine, and tyrosine residues on nuclear, cytoplasmic, and mitochondrial proteins.^1,2^ By utilizing the hexosamine biosynthetic pathway, O-GlcNAcylation links major metabolic pathways to the regulation of multiple cellular processes, including gene expression, signal transduction, and cellular stress responses.^3–7^ Dysregulation of O-GlcNAcylation has been implicated in diverse human diseases, including cancer, where it contributes to tumor initiation, progression, and metabolic reprogramming.^8–16^

Liver cancer, primarily hepatocellular carcinoma (HCC), which accounts for 75%–85% of cases, is the third leading cause of cancer death and the sixth most frequently diagnosed cancer, with 865,000 new cases and 757,948 deaths in 2022.^17^ Of note, elevated levels of O-GlcNAcylation have been observed in HCC tissues compared with adjacent normal liver tissues.^18^ Multiple studies indicate that O-GlcNAcylation on proteins plays a critical role in the pathogenesis of HCC by promoting tumor initiation, growth, invasion, metastasis, and chemoresistance.^19–26^ Despite this progress, the comprehensive profiling of site-specific O-GlcNAcylation in clinical tissues remains technically challenging.

Site-specific and quantitative O-GlcNAc proteomics provide a glimpse of the dynamics of all O-GlcNAcylated proteins and sites, with the potential to elucidate the functional roles of O-GlcNAcylation in health and disease.^27,28^ To date, quantitative O-GlcNAc proteomics has been performed in different types of cells.^29–37^ For example, by integrating metabolic chemoenzymatic labeling and click chemistry (using isotopic photocleavable tags), Li et al. quantified O-GlcNAc proteome changes between sorafenib-sensitive and sorafenib-resistant HepG2 liver cancer cells.^34^ They found increased O-GlcNAcylation on 55 peptides (corresponding to 47 proteins) and decreased O-GlcNAcylation on 136 peptides (corresponding to 105 proteins) in the sorafenib-resistant cells, indicating that altered O-GlcNAcylation is associated with liver cancer chemoresistance. With a similar approach, Liu et al. profiled the O-GlcNAcylation of proteins in colorectal cancer cell lines SW480 and SW620.^37^ They found increased levels of O-GlcNAcylation at 54 O-GlcNAc sites on 41 proteins in SW480 cells and 242 O-GlcNAc sites on 141 proteins in SW620 cells, suggesting that substantially altered levels of O-GlcNAcylation on proteins are involved in colorectal cancer metastasis. Besides cells, several types of tissue samples have also been characterized for quantitative O-GlcNAc proteomics.^32,38–42^ However, quantitative O-GlcNAc proteomics has been tentatively adopted for cancer tissue samples. In a recent study, Song et al. examined O-GlcNAcylated proteins in hepatoblastoma,^43^ a rare malignant primary hepatic tumor in children. By combining an O-GlcNAc antibody bead-based enrichment and isotopic labeling-based quantification, they identified 114 O-GlcNAc sites, among which 17 showed significant changes between hepatoblastoma and normal tissues. Our recent work showed that, in comparison to the antibody-based affinity enrichment, chemoenzymatic labeling/click chemistry-based enrichment methods generally provide much more efficient enrichment for O-GlcNAc peptides.^2,44–46^ Thus, we reasoned that such methods could render enhanced identification and quantification of O-GlcNAc proteins.

Herein, we report a quantitative O-GlcNAc proteomics workflow for the analysis of O-GlcNAcylation by comparing hepatocellular carcinoma (HCC) and normal liver tissues. In our strategy, tissue proteins were subjected to GalT1­(Y289L)-catalyzed chemoenzymatic labeling, click chemistry with a photocleavable biotin-alkyne probe, proteolytic digestion, and neutravidin chromatography, followed by isotopic labeling with tandem mass tags (TMTs). The resulting O-GlcNAc peptides were analyzed by a nanoUPLC-MS/MS system in HCD product-dependent EThcD (HCD-pd-EThcD) mode for O-GlcNAc site mapping and quantification. The quantified O-GlcNAcylation was normalized to protein abundance and compared between HCC and the normal groups. To the best of our knowledge, this study represents the first application of site-specific and quantitative O-GlcNAc proteomic profiling of human HCC tissues. This data set provides new insights into the differential regulation of O-GlcNAcylation in liver cancer and lays the foundation for further exploration of its functional roles in HCC.

## Experimental Section

### Materials and Reagents

1,4-Dithiothreitol (DTT) was purchased from MP Biomedicals (Solon, OH). Iodoacetamide (IAA), urea, 10% Nonidet P-40 Substitute solution (proteomic grade), and recombinant PNGase F (glycerol-free) were ordered from VWR (Radnor, PA). PUGNAc, triethylammonium bicarbonate (TEAB) buffer (1 M, pH 8.5), sodium dodecyl sulfate (SDS), 50% hydroxylamine solution, complete EDTA-free protease inhibitor cocktail tablets, and trypsin from the porcine pancreas were purchased from Sigma-Aldrich (St. Louis, MO). Trypsin/Lys-C mix (mass spectrometry grade) was obtained from Promega (Madison, WI). Benzonase nuclease was from MilliporeSigma (Burlington, MA). Manganese­(II) chloride tetrahydrate was obtained from TCI America (Portland, OR). PC-biotin-alkyne and BTTAA were obtained from Vector Laboratories (Newark, CA). Uridine 5′-diphospho-*N*-acetylazidogalactosamine disodium salt (UDP-GalNAz) was bought from BioChemSyn. High capacity NeutrAvidin agarose, formic acid (FA, LC/MS grade), CuSO_4_ (98%), l-ascorbic acid sodium salt, 1 M HEPES buffer (pH 7.5), FastAP thermosensitive alkaline phosphatase (TSAP), TMT10plex label reagent set (Lot No. ZF391286), high pH reversed-phase peptide fractionation kit, and BCA protein assay kit were obtained from Thermo Fisher Scientific (Waltham, MA). LC/MS grade solutions of 0.1% FA and 0.1% FA in ACN were ordered from Honeywell (Charlotte, NC). RPTOR antibody was purchased from Cell Signaling Technology (no. 2280, Danvers, MA). Protein A/G agarose beads were from Santa Cruz Biotechnology (no. sc−2003, Dallas, TX). Anti-O-linked *N*-acetylglucosamine antibody RL2 was from Abcam (no. ab2739, Dallas, TX). Micro S-TRAP columns were from ProtiFi (Fairport, NY). UVP XX-15L UV Bench Lamp was from Analytik Jena (Upland, CA). GFP-GalT1 (Y289L) was prepared by following the procedure described previously.^48^ Seven liver tumor samples and seven control tissue samples were obtained from the Histopathology and Tissue Shared Resource, Lombardi Comprehensive Cancer Center. All experimental biospecimens were provided under HTSR IRB 1992-048 in a deidentified manner (with details shown in the Supporting Information Table S1).

### Protein Extraction

Tissue samples were rinsed with cold PBS and minced on ice. Lysis was performed by adding 0.5 mL of cell lysis buffer (5% SDS, 75 mM NaCl, 1 mM EDTA, 50 μM PUGNAc, 1× protease inhibitor cocktail, 50 mM TEAB, 5 μL of benzonase, and 5 mM MgCl_2_), followed by homogenization using a Raptor tissue grinder. Samples were then sonicated on ice with a probe-tip sonicator for three cycles (10 s on and 20 s off per cycle). The lysates were centrifuged at 14,000 g for 15 min at 4 °C, and the supernatants were collected. Protein concentrations were determined using the BCA assay.

### Total Protein Analysis

Total protein analysis was performed using micro S-TRAP columns, similar as described previously.^47^ Briefly, 20 μg of proteins extracted from the whole lysate in 5% SDS were reduced with 20 mM DTT, alkylated with 40 mM IAA, acidified with 10% TFA, and diluted with the binding/wash buffer (100 mM TEAB, pH 7.55, in 90% methanol). The diluted samples were loaded onto the micro S-TRAP columns and washed five times with the binding/wash buffer. Subsequently, 1 μg of trypsin/Lys-C mix in 50 mM TEAB was added, and the samples were incubated at 37 °C overnight. The resulting peptides were eluted sequentially with 50 mM TEAB, 0.2% aqueous formic acid, and 50% ACN. Finally, the combined eluates were lyophilized and resuspended in 0.1% formic acid for nanoUPLC-MS/MS analysis.

### O-GlcNAc Proteomics

Four milligrams of extracted proteins from the whole lysate were used for O-GlcNAc enrichment, following a procedure similar to that described previously.^2,46^ Briefly, proteins were reduced with 20 mM DTT and alkylated with 40 mM IAA, followed by precipitation using chloroform, methanol, and water. The resulting precipitate was dissolved in 400 μL of 1% SDS in 20 mM HEPES buffer (pH 7.9) and incubated at 95 °C for 5 min. Chemoenzymatic labeling was performed at the protein level by adding 490 μL of H_2_O, 800 μL of labeling buffer (50 mM HEPES pH 7.9, 125 mM NaCl, 5% NP-40), 105 μL of 100 mM MnCl_2_, 100 μL of 0.5 mM UDP-GalNAz, 10 μg of GalT1­(Y289L), 0.5 μL of PNGase F, and 0.5 μL of TSAP and incubating the mixture overnight at 4 °C. Labeled proteins were then precipitated and resuspended in 1 mL of 0.5% SDS with 20 mM HEPES (pH 7.9). The CuAAC reaction was performed by adding 11 μL of 10 mM PC-biotin-alkyne, 33 μL of 10 mM CuSO_4_–BTTAA (1:2), and 55 μL of 50 mM sodium ascorbate, followed by incubation at room temperature for 2 h. After precipitation, proteins were resuspended and digested with 150 μg of trypsin in 1.6 M urea and 50 mM HEPES (pH 7.9). The peptide mixture was incubated with high-capacity NeutrAvidin beads in PBS for 3 h at room temperature. Finally, the conjugated peptides were released in 0.1% formic acid by irradiation at 365 nm for 1 h. The released peptides were dried and resuspended in 50 μL of 50 mM TEAB buffer (pH 8.5) for labeling with 0.1 mg of TMT10plex reagent, following the manufacturer’s protocol. Tumor samples were assigned to the N channels, and normal samples were assigned to the C channels. Labeled samples were combined at a 1:1 ratio and fractionated using the Pierce high-pH reversed-phase peptide fractionation kit according to the vendor instructions. Eight fractions were collected, dried, and reconstituted in 0.1% formic acid for nanoUPLC-MS/MS analysis.

### NanoUHPLC-MS/MS

Peptides from total tissue lysate without enrichment were analyzed using a trapped ion mobility-quadrupole time-of-flight mass spectrometer (timsTOF Ultra 2, Bruker Daltonics, Bremen, Germany). Sample separation was performed on a nanoElute 2 nanoflow ultrahigh-performance liquid chromatography (UHPLC) system equipped with a PepSep ULTRA C18 column (15 cm × 75 μm, 1.5 μm) at 50 °C and a flow rate of 300 nL/min (Bruker Daltonics). The binary gradient consisted of mobile phase A (water with 0.1% formic acid) and mobile phase B (ACN with 0.1% formic acid) as follows: 2% B at 0 min, 23% B at 28 min, 30% B at 32 min, 90% B at 36 min, and 90% B at 40 min. The nanoLC was coupled to a timsTOF Ultra 2 mass spectrometer via a nanoelectrospray ion source (CaptiveSpray, Bruker Daltonics) operated in dia-PASEF mode with positive polarity. Singly charged precursors were excluded by a polygon filter. The capillary voltage was set to 1,600 V, with a dry gas flow rate of 3 L/min and a dry temperature of 200 °C. Mass spectra were acquired over an *m*/*z* range of 100 to 1,700, with ion mobility scanned from 0.64 to 1.45 (V·s)/cm^2^. Precursors for data-independent acquisition were isolated with ion mobility-dependent collision energy, linearly increased from 20 to 59 eV. The total acquisition cycle was 1.70 s, consisting of one MS1 ramp and 20 MS/MS ramps (60 windows), covering the range of 350.7 to 1,250.6 Da.

A nanoACQUITY UPLC system (Waters, Milford, MA) coupled to an Orbitrap Fusion Lumos mass spectrometer (Thermo Fisher Scientific, Waltham, MA) was used to analyze the fractions of enriched O-GlcNAc peptides, using instrument settings similar to those described previously.^2,46,48^ Samples were loaded onto a C18 trap column (Waters Acquity UPLC M-Class Trap, Symmetry C18, 100 Å, 5 μm, 180 μm × 20 mm) at 10 μL/min for 4 min and then separated on an analytical column (Waters Acquity UPLC M-Class, Peptide BEH C18, 300 Å, 1.7 μm, 75 μm × 150 mm) at a column temperature of 45 °C and a flow rate of 400 nL/min. A 150 min gradient was used with buffer A (0.1% FA in 2% ACN) and buffer B (0.1% FA in ACN) as follows: 1% buffer B at 0 min, 5% buffer B at 1 min, 22% buffer B at 105 min, 36% buffer B at 125 min, 50% buffer B at 130 min, 90% buffer B at 135 min, 90% buffer B at 145 min, 1% buffer B at 145.1 min, and 1% buffer B at 150 min. Data-dependent acquisition (DDA) was used to acquire MS data with an ion spray voltage of 2.6 kV and an ion transfer temperature of 275 °C. The MS parameters were set as follows: Detector Type: Orbitrap; Orbitrap Resolution: 120,000; Scan Range: *m*/*z* 350–1,800; RF Lens: 30%; AGC Target: Standard; Maximum Injection Time Mode: Auto; Microscans: 1; Charge State: 2–8; Cycle Time: 3 s. HCD product-dependent EThcD (HCD-pd-EThcD) with a dynamic exclusion duration of 40 s was applied for the MS/MS acquisition. EThcD was triggered by the oxonium ions of HexNAc (*m*/*z* 126.055, 138.055, 144.066, 168.065, 186.076, and 204.086) as well as the major fragments resulting from the alkyne tag (*m*/*z* 300.130, 503.210, 529.293, and 732.372) observed in HCD scans. MS/MS parameters were set as follows: Isolation Mode: Quadrupole; Isolation Window: *m*/*z* 1.6; HCD Collision Energy : 30%. Detector Type: Orbitrap; Resolution: 50,000; AGC Target: Standard. Supplemental Activation (SA) collision energy of EThcD was set at 30%.

### Data Analysis

DIA data for label-free proteomics acquired on the timsTOF Ultra 2 mass spectrometer were analyzed with Spectronaut v20.0 (Schlieren, Switzerland) using the directDIA workflow without a spectral library against the UniProt *Homo sapiens* database (TaxID 9606, downloaded on February 22, 2023, 20,404 sequences) with default settings. Briefly, acetyl at the protein N-terminus and oxidation on M were set as variable modifications, and a maximum of five variable modifications were allowed. Carbamidomethyl on C was set as a fixed modification. Trypsin/P was set as the enzyme for digestion, allowing for at most two missed cleavages. The FDR was 0.01 on the PSM, peptide, and protein group levels. Precursor identification was required in at least 50% of runs, and missing values were imputed using the background signal.

Raw data of enriched O-GlcNAc peptide fractions acquired on an Orbitrap Fusion Lumos mass spectrometer were analyzed with Proteome Discoverer v2.4 (Fisher Scientific) with the Sequest search engine against the UniProt *Homo sapiens* database (TaxID 9606, downloaded on February 22, 2023, 20,404 sequences). The mass tolerances for the precursor and fragment were set at ± 10 ppm and ± 0.02 Da, respectively. Trypsin/P was set as the enzyme for digestion, allowing for at most two missed cleavages. TMT10plex (+229.163 Da) on Lys and N-terminus of peptides and carbamiodomethyl (+57.021 Da) on cysteine were set as static modifications. Mass addition of TMT-labeled AMTzHexNAc2 (+731.364 Da) on Ser/Thr/Tyr/Asn was set as dynamic modification as well as asparagine deamidation (+0.984 Da), methionine oxidation (+15.995 Da), protein N-terminal acetylation (+42.011 Da), N-terminal methionine loss (−131.040 Da), and N-terminal methionine loss plus acetylation (−89.030 Da). The modification sites identified were further filtered by IMP-ptmRS with a site probability ≥0.75. Percolator was used for validation with a target-decoy strategy. The FDRs for peptide-spectrum matches (PSMs), peptide groups, and protein groups were 0.005, 0.009, and 0.023, respectively.

For reporter ion quantification, HCD was selected as the activation type. S/N was used for reporter quantification peaks if all spectrum files had S/N values. Otherwise, the intensities were used. The quantification value was corrected for isotopic impurity following the reporter ion isotopic distributions provided by the vendor. The co-isolation threshold was set at 50%. The average reporter S/N threshold was set at 10. The SPS mass match percentage threshold was set at 65%. Within-group quantile normalization was performed across all of the peptides. Abundances of O-GlcNAc peptides were normalized to the corresponding protein levels obtained from total proteome analysis. For proteins with missing values, a second-stage imputation was performed using the median abundance within the same biological group prior to normalization. If protein data were entirely missing across all samples, the original O-GlcNAc peptide values were retained.

The R packages clusterProfiler,^49^ org.Hs.eg.db,^50^ enrichplot,^51^ ggfortify,^52^ and tidyverse^53^ were used for gene ontology (GO) enrichment analysis, principal component analysis, and data visualization. Statistical comparisons were assessed using *t* tests with the Benjamini–Hochberg adjustment. Protein–protein interactions were retrieved from the STRING v12.0 database at a combined score ≥400,^54^ and the resulting network was partitioned in Cytoscape^55^ using the GLay community-clustering algorithm.^56^ The top GO Biological Process term with the lowest adjusted p-value was selected to represent each cluster using BiNGO in Cytoscape.^57^

### Immunoprecipitation and Immunoblotting

Proteins from three pairs of tissue samples were extracted using a lysis buffer containing 50 mM Tris-HCl (pH 8), 200 mM NaCl, 5 mM EDTA, 0.5% NP-40, 0.5% SDS, 50 μM PUGNAc, and 1x protease inhibitor cocktail. Cleared cell lysates were incubated with the RPTOR antibody and protein A/G agarose beads at 4 °C overnight. After washing with the lysis buffer, bound proteins were analyzed by Western blotting assays. Protein levels and the O-GlcNAcylation levels were blotted with the RPTOR antibody and RL2, respectively.

## Results and Discussion

### Experimental Rationale

Given that protein levels may contribute to altered O-GlcNAcylation,^29,39,40^ we performed both quantification of total proteins and quantification of O-GlcNAc peptides (Figure 1). An equal amount of proteins (20 μg) from each sample was used for quantitative proteomics in label-free data-independent acquisition mode, similar to those described previously.^47,58^ Regarding O-GlcNAc proteomics, 4 mg of proteins from each sample was subjected to GalT1­(Y289L)-mediated chemoenzymatic labeling and photocleavable biotin-alkyne-based enrichment,^46^ with enriched O-GlcNAc peptides labeled with TMT10plex reagents. To maximize O-GlcNAc coverage, the TMT-labeled peptides were pooled and then fractionated into eight fractions using high-pH reversed-phase chromatography, with each fraction analyzed by nanoUPLC-MS/MS in HCD-pd-EThcD mode for O-GlcNAc site mapping and reporter ion-based quantification. Of note, PC-alkyne-biotin, used in the chemoenzymatic enrichment workflow, leaves behind a primary amine-containing tag at the O-GlcNAcylated residue after UV cleavage. This amine group reacts with TMT reagents, similar to native lysine side chains and peptide N-termini; however, only labeling of this amine group results in a +731.364 Da mass addition, which serves as a diagnostic signature for the modified residues.

**Figure 1:**
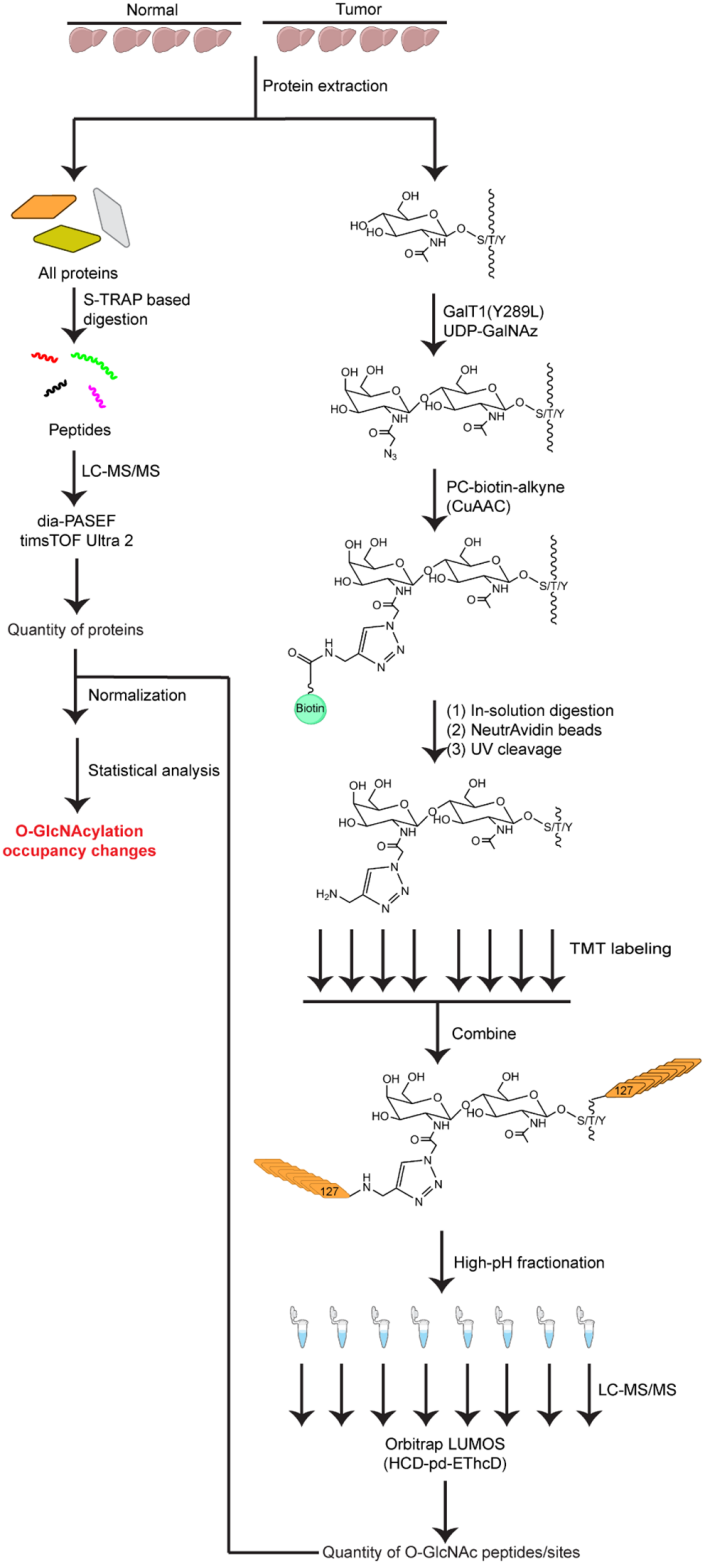
Experimental workflow for quantitative proteomics analysis of O-GlcNAcylation in HCC and normal liver tissues.

The quantified O-GlcNAcylation was normalized to protein abundance and compared between HCC and normal groups using the following equation:

O‐GlcNAcsiteoccupancybetweenHCCandnormaltissues=[O‐GlcNAclevelsinHCC/proteinlevelsinHCC]/[O‐GlcNAclevelsincontrol/proteinlevelsincontrol]=[O‐GlcNAclevelsinHCC/O‐GlcNAclevelsincontrol]/[proteinlevelsincontrol/proteinlevelsinHCC]

similar to what we did previously.^39,40^ It is noteworthy that, although the enriched and nonenriched samples were analyzed using different fragmentation and quantitative modes, all sample preparation procedures, instrument settings, and data-processing parameters were kept identical across samples within each type, i.e., all nonenriched samples were processed and analyzed identically for total proteomics and all enriched samples were processed and analyzed identically for O-GlcNAc proteomics. Since ratios derived from two data sets were used to calculate site-specific O-GlcNAc changes using the aforementioned equation, we reasoned that using two different approaches would not substantially affect the relative O-GlcNAc occupancy estimates.

### Profiling of O-GlcNAcylation in HCC and Normal Tissues

Liver tissue samples from two groups—four HCC and four normal tissues—were compared using the workflow illustrated in Figure 1. Proteins were extracted from liver tissues and first subjected to quantitative proteomics analysis. In total, 6,888 protein groups were identified and quantified across samples (Table S2).

Among the protein groups, 683 proteins were increased and 195 were decreased (i.e., with an absolute fold change ≥ 1.5 and an adjusted *p* value <0.05; Supporting Information Figure S1; Table S2). GO enrichment analysis suggests that significantly increased proteins are primarily associated with RNA splicing and ribosome regulation (Supporting Information Figure S2). In contrast, significantly decreased proteins are mainly involved in amino acid metabolic processes and others (Supporting Information Figure S2).

For O-GlcNAc profiling, among the 1,917 peptides identified across all samples, 602 peptides contained at least one occurrence of the characteristic mass shift (i.e., +731.364 Da mass addition) (). Enrichment specificity was highly similar between tumor (31.4%) and control samples (31.3%; Welch *t* test, p = 0.057), and the numbers of identified glycopeptides were also comparable (602 in tumor vs 598 in control; Welch *t* test, p = 0.016). Across all samples, 602 peptides with a mass addition of 731.364 Da were identified, and only 10 were not detected in every sample, indicating negligible enrichment bias. By filtering for modifications on Ser, Thr, and Tyr residues, 440 O-GlcNAc peptides were identified (Table S3). In total, 305 O-GlcNAcylation sites from 196 proteins were confidently assigned, each with a localization probability >94% (Table S4).

Remarkably, in comparison to the O-GlcNAcylated proteins identified in hepatoblastoma in which O-GlcNAc antibody beads were used for enrichment,^43^ we newly identified 291 O-GlcNAcylated sites on proteins from HCC samples. Furthermore, many transcriptional coactivators and transcription factors, which are generally of low abundance and usually underrepresented in proteome profiling experiments, were found to be O-GlcNAcylated using our strategy. For example, O-GlcNAcylation was identified on the protein nuclear receptor coactivator 6 (NCOA6), a regulator which plays a fundamental role in transcriptional activation and HCC development.^59,60^ Figure 2 shows the representative mass spectra of the two O-GlcNAcylated sites (i.e., S1641 and T1933). Besides transcriptional coactivators, O-GlcNAcylation changes on a number of transcription factors were also revealed. For example, S1550 and S1864 on Zinc finger protein 40 (HIVEP1) were found O-GlcNAcylated (Supporting Information Figure S3).

**Figure 2:**
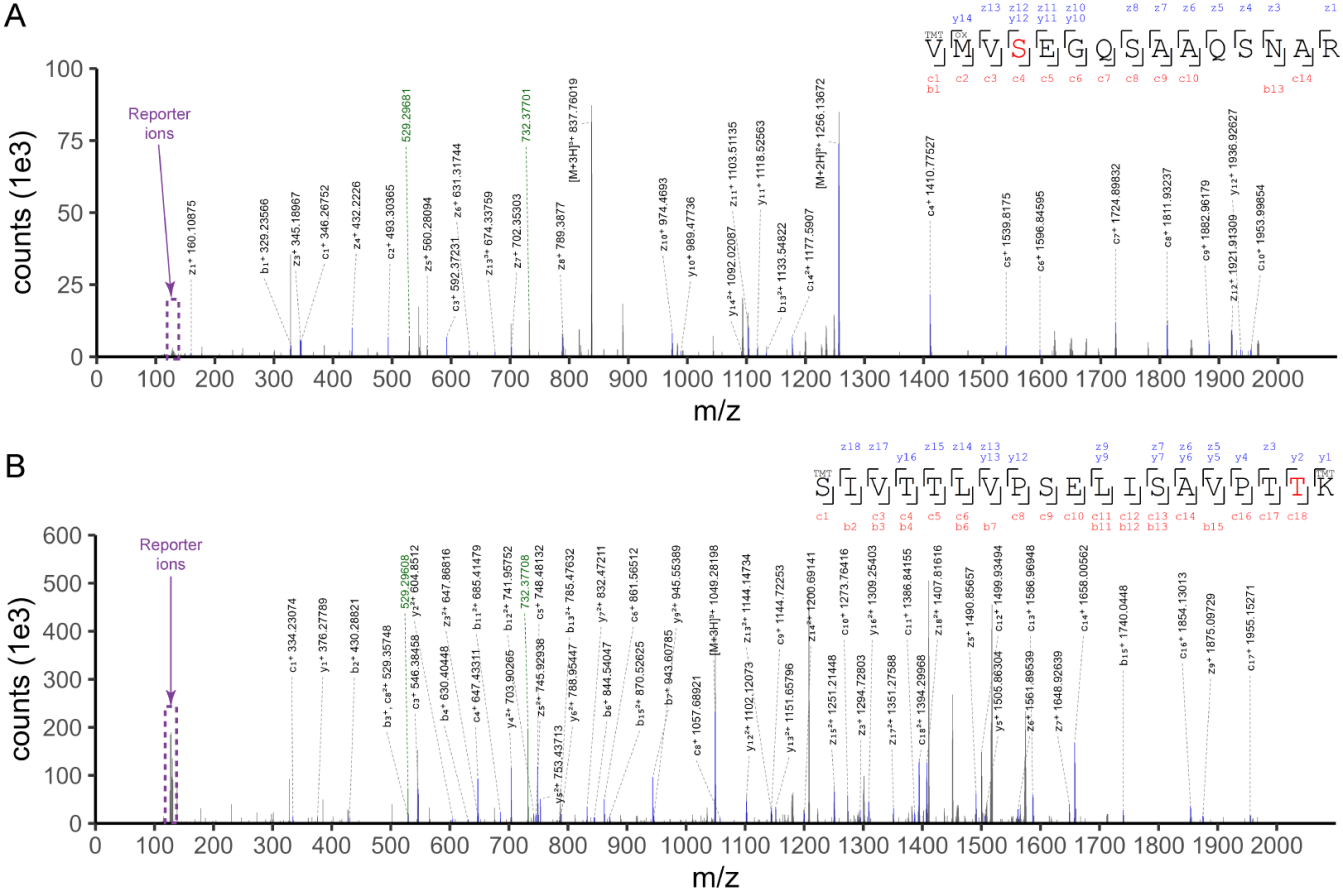
Representative mass spectra of two O-GlcNAcylated sites, i.e., S1641 (A) and T1933 (B), on nuclear receptor coactivator 6 (NCOA6). Matched b, y, c, and z ions are annotated, with modified amino acids shown in red and the peptide N-term TMT labeled. Two key fragments resulting from the PC-biotin-alkyne tag, i.e., *m*/*z* 732.37 and 529.29, are highlighted in green. TMT reporter ions are shown in purple.

### Altered O-GlcNAc Site Occupancy between HCC and Normal Tissues

Among the 440 identified O-GlcNAc peptides, 390 were successfully quantified using TMT reporter ion intensities, allowing a robust comparison of the patterns of O-GlcNAcylation between HCC and normal liver tissues. The abundances of O-GlcNAc peptides were first normalized across samples within each group using quantile normalization and then further adjusted to the corresponding protein levels obtained from total proteome quantification. After log_2_ transformation, the normalized quantities of O-GlcNAc peptides from all samples were subjected to principal component analysis (PCA). HCC and normal samples clustered separately using a t-distribution-based confidence ellipse (Figure 3A), indicating substantial global changes in the O-GlcNAcylation landscape associated with malignancy. This separation suggests that O-GlcNAcylation may contribute to, or reflect, underlying molecular differences between tumor and normal tissues.

**Figure 3:**
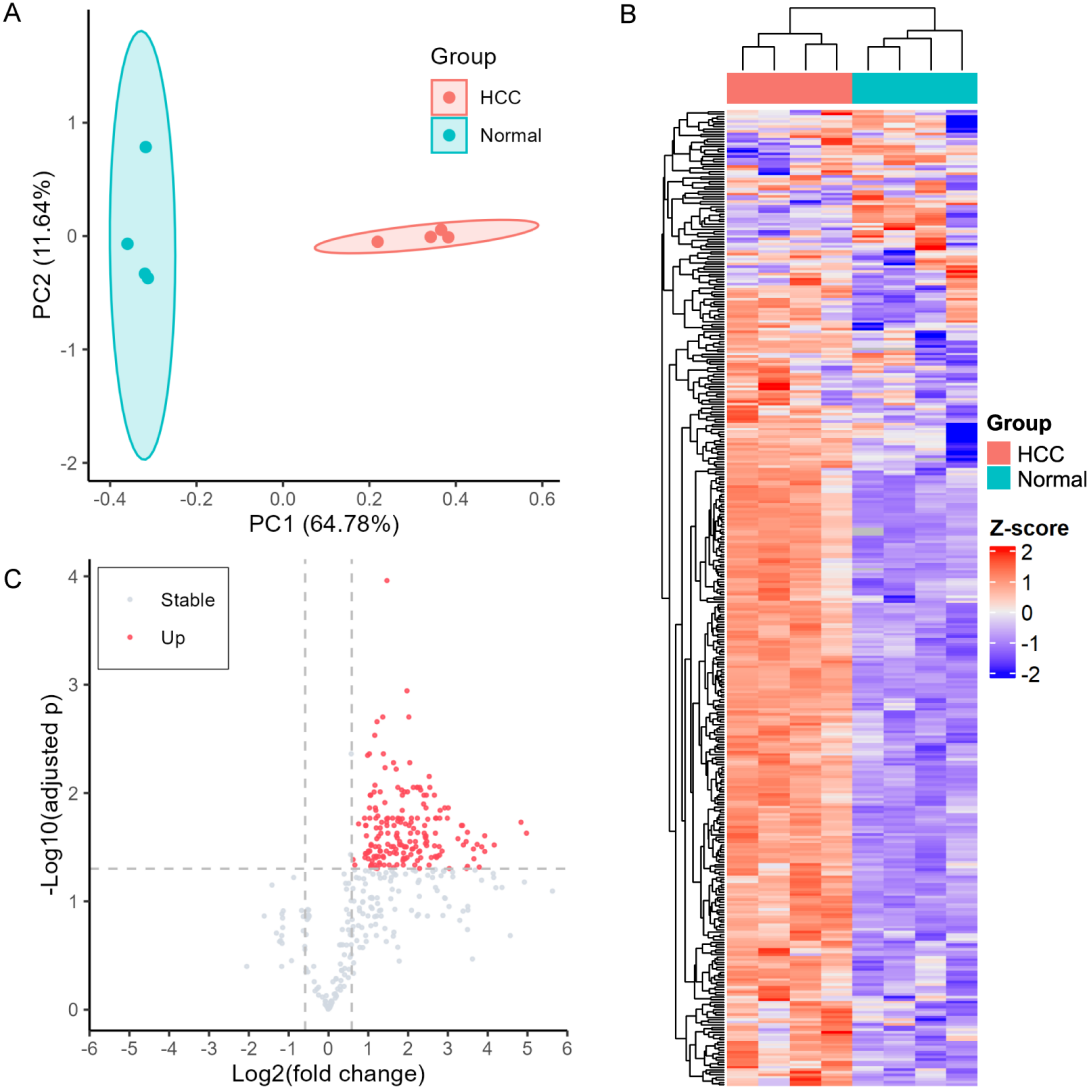
Quantitative analysis of O-GlcNAcylation in HCC and normal liver tissues: (A) PCA analysis; (B) hierarchical clustered heatmap; (C) volcano plot.

To further explore patterns of differential O-GlcNAcylation, we generated a heatmap using Z-scored O-GlcNAc quantities (Figure 3B). As seen, O-GlcNAcylation was elevated in HCC with samples clustering by biological groups. Despite the limited number of biological replicates in our study, the widespread increase in O-GlcNAcylation aligns with the findings of Zhu et al.,^18^ who reported that global O-GlcNAcylation levels are markedly higher in HCC tissues compared to healthy control liver tissues and significantly elevated in recurrent HCC tissues compared to recurrence-free tissues, based on an immunohistochemistry assay for dozens of samples.

O-GlcNAc sites between the two groups were assessed using fold change and *t* test p values, which were adjusted for multiple comparisons using the Benjamini–Hochberg method. A complete list of the quantified O-GlcNAc peptides is provided in Table S5. Among them, 190 O-GlcNAc peptides from 121 proteins were significantly upregulated in the HCC group, with a fold change ≥ 1.5 and an adjusted *p* value <0.05 (Figure 3C; Table S5). For example, we identified O-GlcNAcylation on T700 of the regulatory-associated protein of mTOR (RPTOR), a core component of the mechanistic target of rapamycin complex 1 (mTORC1)^61,62^ (Figure 4A). Although there was only a slightly over 1.5-fold increase in the protein level (Figure 4B), up to an 8.5-fold higher level of O-GlcNAcylation on RPTOR was found in HCC (in comparison to control samples) (Figure 4C). After normalization to the protein level change, an ∼5.3-fold higher O-GlcNAc site occupancy was observed on T700 of RPTOR (Figure 4D). To further confirm and validate our findings, we immunoprecipitated RPTOR from three other pairs of normal and HCC lysates. Similarly, substantially higher levels of O-GlcNAcylation on RPTOR were found in HCC (in comparison to control samples), despite the comparable protein abundances (Figure 4E). Collectively, these data show upregulated O-GlcNAcylation on RPTOR across HCC samples. Consistently, a recent study revealed that T700 of RPTOR is O-GlcNAcylated in HEK293T when RPTOR is coexpressed with OGT under glucose sufficiency.^63^ More importantly, O-GlcNAcylation on T700 of RPTOR enhances its interaction with Rag GTPases, promoting mTOR translocation to the lysosomal surface and thereby activating mTORC1.^63^ Thus, O-GlcNAcylation on RPTOR is critical for regulating mTORC1, a master nutrient sensor that responds to signals like amino acids and glucose and orchestrates cellular growth. In addition, both O-GlcNAcylation and mTORC1 are closely involved in multiple types of cancers.^8–10,12,13,15,16,64,65^ Taken together, these data suggest that it would be very intriguing to explore whether and how dysregulated RPTOR O-GlcNAcylation contributes to HCC development.

**Figure 4:**
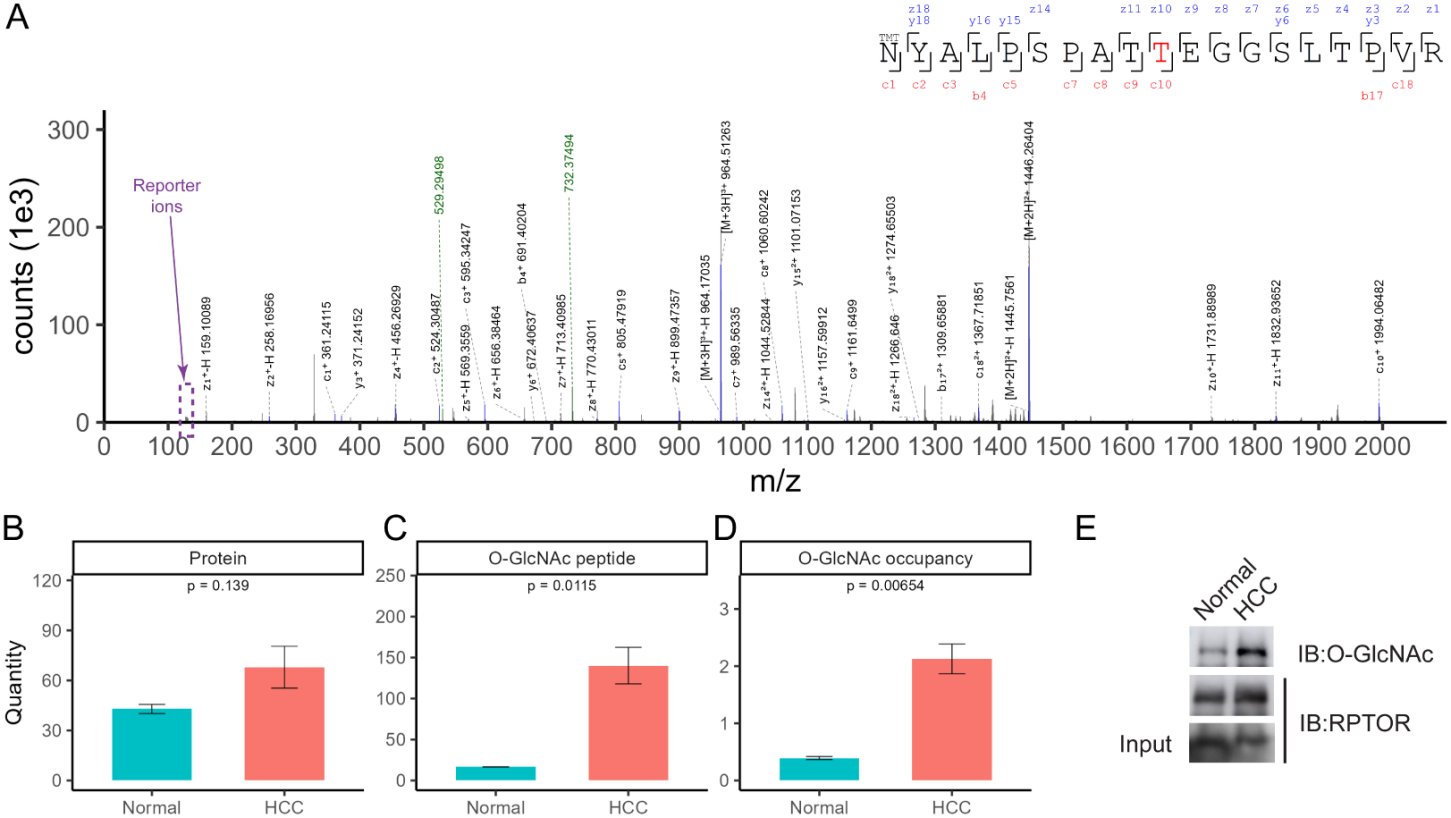
(A) Representative mass spectrum of O-GlcNAcylation on T700 of the regulatory-associated protein of mTOR (RPTOR), quantification of (B) the protein level, (C) the O-GlcNAcylation level, (D) the O-GlcNAc T700 site occupancy of RPTOR, and (E) Western blotting of O-GlcNAcylation of RPTOR in HCC and control tissue lysates (*n* = 3). Of note, the O-GlcNAcylated Thr in the peptide is highlighted in red, and the N-term is TMT labeled. Two key fragments resulting from the tag, i.e., *m*/*z* and 732.37 and 529.29, are highlighted in green. TMT reporter ions are shown in purple.

The exclusive upregulation of the occupancy of the O-GlcNAc site on proteins emphasizes the potential functional relevance of this modification in HCC pathogenesis and highlights the role of O-GlcNAcylation as a promising target for therapeutic intervention and biomarker discovery.

### Functional Classification of Altered O-GlcNAc Proteins

We performed gene ontology (GO) and KEGG pathway enrichment analyses based on the 121 proteins that exhibited significantly increased O-GlcNAcylation to gain insights into their biological significance (Figure 5). Proteins exhibiting up-regulated O-GlcNAcylation are predominantly associated with nuclear-related biological processes, including nuclear import, nucleocytoplasmic transport, nuclear pore organization, and others. O-GlcNAcylated proteins are also associated with nuclear inclusion bodies, nuclear pore complexes, and the nuclear membrane. In addition, these proteins are highly involved in molecular functions, such as nuclear transport, transcription regulation, nuclear receptor interactions, signal sequence recognition, and transcription coactivation.

**Figure 5:**
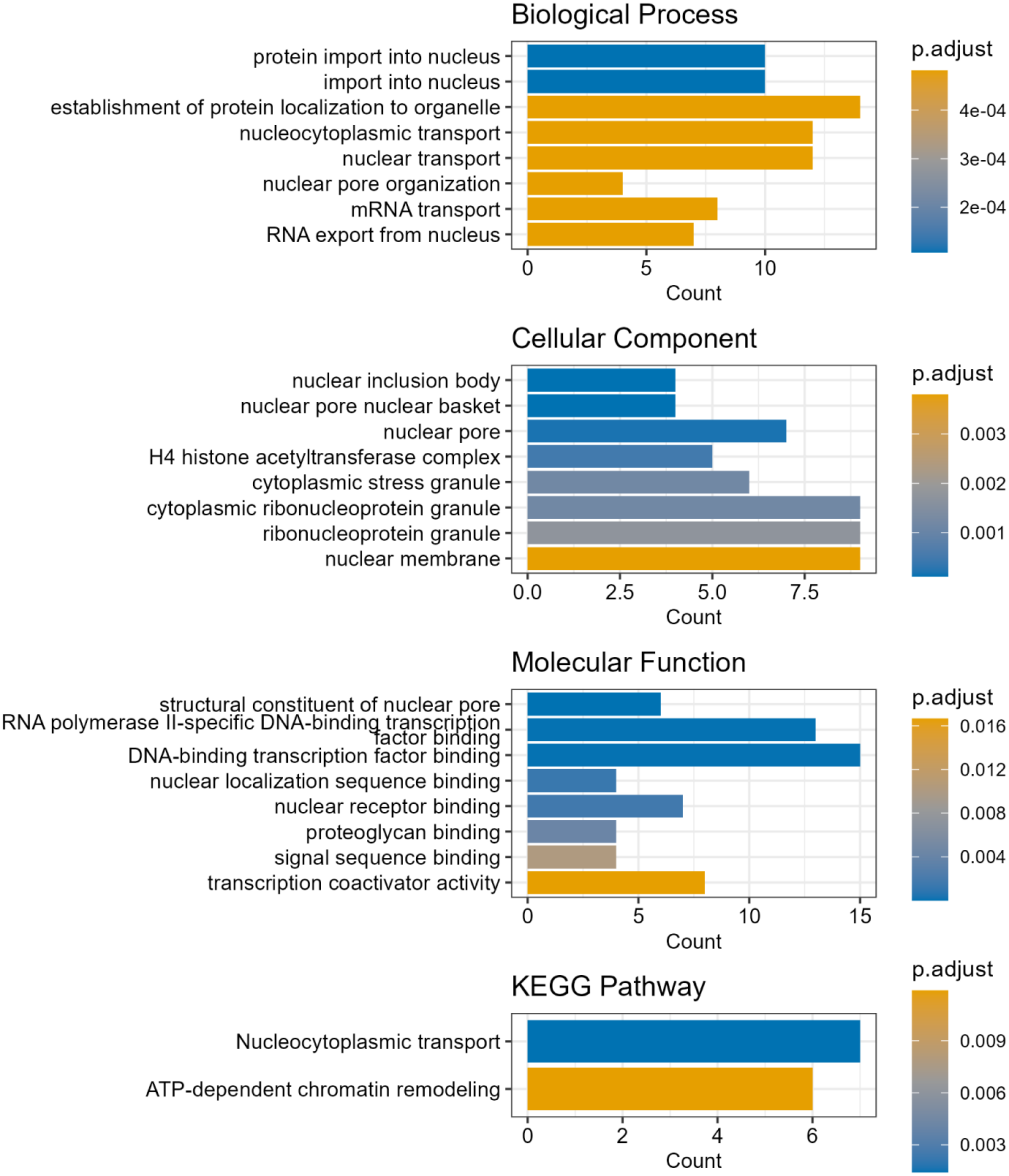
Top eight GO terms and KEGG pathways enriched among proteins with significantly increased levels of O-GlcNAcylation.

KEGG pathway enrichment highlighted the involvement in nucleocytoplasmic transport and ATP-dependent chromatin remodeling. It is known that ATP-dependent chromatin remodeling is critical to regulate gene expression and is closely associated with cancer development.^66^ Thus, elucidating the roles of the O-GlcNAcylated proteins in chromatin remodeling may provide invaluable clues to understand HCC growth, metastasis behavior, stemness features, and therapeutic resistance.

To further explore the potential coordination and functional interplay among these O-GlcNAcylated proteins, we constructed a functionally clustered protein–protein interaction (PPI) network using high-confidence interactions in the STRING database v12.0 at a combined score ≥ 400 with GLay partitioning (Figure 6). The resulting network revealed seven densely connected communities comprising 74 genes in total. GO biological process terms with the lowest adjusted *p*-value were selected to represent the six communities (Figure 6). The result shows that these proteins are highly involved in the negative regulation of gene expression, chromatin modification, regulation of translation, mRNA transport, and ER to Golgi vesicle-mediated transport. Many of the O-GlcNAc proteins (e.g., STAT3, RPTOR, and NCOR1) may interact with each other, indicating a potential synergistic role in signal transduction and transcriptional regulation in HCC development

**Figure 6:**
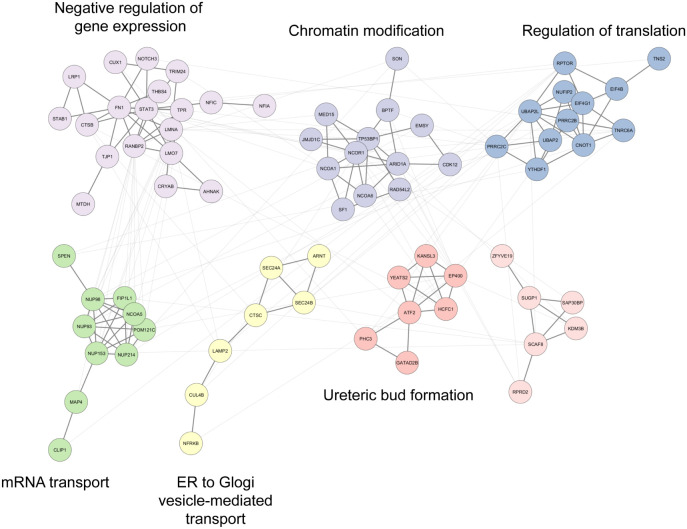
Clustered protein–protein interaction network of 74 proteins displaying significantly increased levels of O-GlcNAcylation with representative GO biological process terms.

### Characterization of N-GlcNAcylation

Previously, we found that the chemoenzymatic labeling approach allows detection of N-linked *N*-acetyl-d-glucosamine monosaccharide (*N*-GlcNAc) modification on asparagine (Asn, N) residues of some proteins.^46^ Thus, we checked the presence of N-linked GlcNAc peptides/sites in our data set. In total, 152 Asn residues on 118 proteins were modified by GlcNAc with high confidence (localization probability ≥ 75%; Table S6). Similar to our previous observation,^46^ a vast majority of Asn residues have been reported to be N-glycosylation sites for glycans, according to UniProt.^67^ Very unexpectedly, after normalizing to protein levels, ten N-GlcNAc sites were found significantly changed between control and HCC samples (Table S7). Specifically, eight N-GlcNAc sites were significantly upregulated in the HCC group. For example, we identified GlcNAcylation on N1067 of Fibrillin-1 (FBN1) (Supporting Information Figure S4A), an essential component of the extracellular matrix.^68,69^ Of note, despite the ∼40% decreased protein level, an ∼2.4-fold increased N-GlcNAcylation on N1067 was identified in HCC. Concomitantly, an ∼4.3-fold increased GlcNAc site occupancy on N1067 was obtained (Supporting Information Figure S4B). Interestingly, although GlcNAcylation also modified two other sites (i.e., N448 and N1581) of FBN1, no significant changes were observed, suggesting differential N-GlcNAcylation occupancy on multiple sites of a given protein. Besides the significantly upregulated *N*-GlcNAc sites and largely stable *N*-GlcNAc sites, two *N*-GlcNAc sites were significantly downregulated in the HCC group (i.e., N74 of Carboxypeptidase N subunit 2 and N282 of arylacetamide deacetylase). N-GlcNAcylation has been considered enigmatic for some time as it had no known outcome or association.^70–72^ As the first of its kind quantitative *N*-GlcNAc proteomics study, our work indicates a potential role of N-GlcNAcylation on proteins in HCC development, while its functional importance in physiology and pathology awaits to be elucidated.

## Conclusions

In this study, we applied an integrated approach by coupling chemoenzymatic enrichment and a TMT-based quantitative proteomics approach to characterize the O-GlcNAcylation landscape in hepatocellular carcinoma. Our workflow identified 440 O-GlcNAc peptides, representing 305 sites on 196 proteins. Among them, 190 O-GlcNAc peptides from 121 proteins were significantly upregulated in HCC. Functional annotation and protein–protein interaction mapping of these proteins underscored their involvement in processes such as signal transduction, nucleocytoplasmic transport, and transcriptional regulation. This comprehensive data set offers new insights into the oncogenic role of O-GlcNAcylation and represents a valuable foundation for mechanistic and translational research for liver cancer.

## Supplementary Material

















## Data Availability

All mass spectrometry data files have been deposited to the MassIVE data repository under accession number MSV000099254.

## Notes

Volcano plot of proteins quantified in HCC and normal liver tissues (Figure S1); GO enrichment of significantly changed proteins between HCC and normal liver tissues (Figure S2); representative mass spectra of two O-GlcNAcylated sites, i.e., S1550 (A) and S1864 (B) on zinc finger protein 40 (HIVEP1) (Figure S3); (A) representative mass spectrum of GlcNAcylation on N1067 of Fibrillin-1 (FBN1) and (B) quantification of the protein level, N1067 GlcNAcylation level, and GlcNAc site occupancy on N1067 of FBN1 (Figure S4) (PDF)
Information of the liver samples used (Table S1) (XLSX)
List of all proteins identified and quantified (Table S2) (XLSX)
List of all peptides identified in O-GlcNAc proteomics (Table S3) (XLSX)
List of unambiguous O-GlcNAc sites identified (Table S4) (XLSX)
List of O-GlcNAc site occupancy changes between HCC and normal samples (Table S5) (XLSX)
List of unambiguous *N*-GlcNAc sites identified (Table S6) (XLSX)
List of *N*-GlcNAc site occupancy changes between HCC and normal samples (Table S7) (XLSX)
