# Supplementary material for: Site-Specific and Quantitative O‑GlcNAc Proteomics for Hepatocellular Carcinoma
Source: J Proteome Res. 2026 Mar 11;25(4):1892–902. doi: 10.1021/acs.jproteome.5c00939 (PMC13054871; doi:10.1021/acs.jproteome.5c00939)
Supplement: Supplementary file 1 [file pr5c00939_si_001.pdf]

Supporting Information

Site-specific and Quantitative O-GlcNAc Proteomics for Hepatocellular Carcinoma

Chunyan Hou,[1#] Ping Li[1,2#], Ethan Pei,[1] Hemeng Zhang,[1] Ci Wu,[1] Jingtao Deng,[1] Stephen W. Byers,[1] Junfeng Ma[1,*]

[1] Department of Oncology, Lombardi Comprehensive Cancer Center, Georgetown University Medical Center, Washington DC 20007, USA

[2] Department of Oncology, Renmin Hospital of Wuhan University, Wuhan 430064, China

[#] Equal contribution.

[*] To whom correspondence should be addressed: Tel: +1-202-6873802; e-mail: junfeng.ma@georgetown.edu

Table of Contents

**Description**	**Page No.**
**Figure S1**. Volcano plot of proteins quantified in HCC and normal liver tissues.	S-3
**Figure S2**. GO enrichment of signiciantly changed proteins between HCC and normal liver tissues.	S-4, S5
**Figure S3.** Representative mass spectra of two O-GlcNAcylated sites, i.e., S1550 (A) and S1864 (B) on Zinc finger protein 40 (HIVEP1).	S-6
**Figure S4.** (A) Representative mass spectrum of GlcNAcylation on N1067 of Fibrillin-1 (FBN1) and (B) quantitation of the protein level, N1067 GlcNAcylation level, and GlcNAc site occupancy on N1067 of FBN1.	S-7
**Table S1.** Information of the liver samples used.	
**Table S2**. List of all proteins identified and quantified.	
**Table S3**. List of all peptides identified in O-GlcNAc proteomics.	
**Table S4**. List of unambiguous O-GlcNAc sites identified.	
**Table S5.** List of O-GlcNAc site occupancy changes between HCC and normal samples.	
**Table S6**. List of unambiguous N-GlcNAc sites identified.	
**Table S7.** List of N-GlcNAc site occupancy changes between HCC and normal samples.	

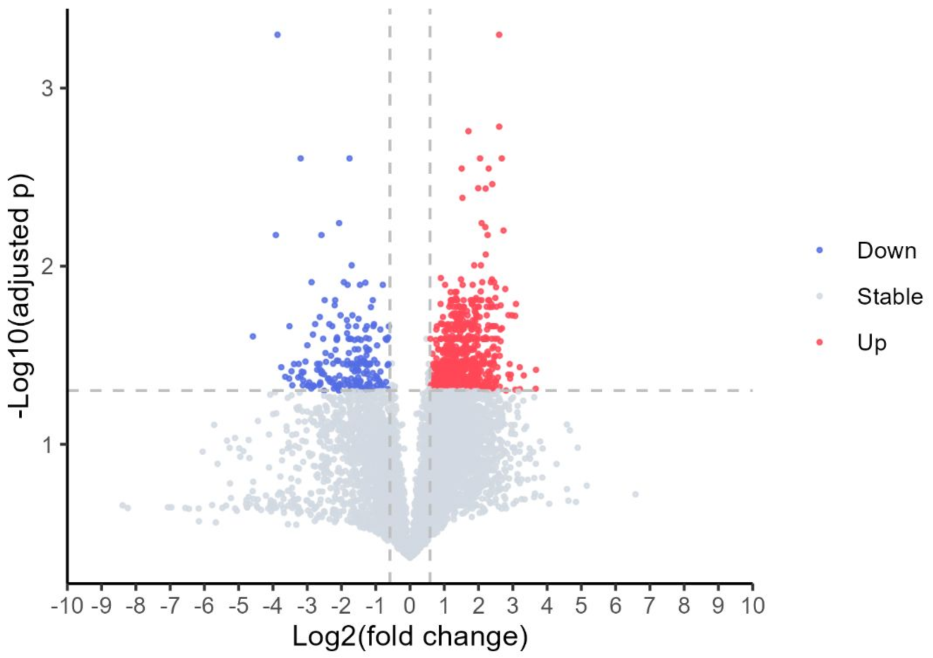

Figure S1. Volcano plot of proteins quantified in HCC and normal liver tissues. Significantly altered proteins are highlighted in blue (decreasd) and red (increased).

Up-regulated proteins

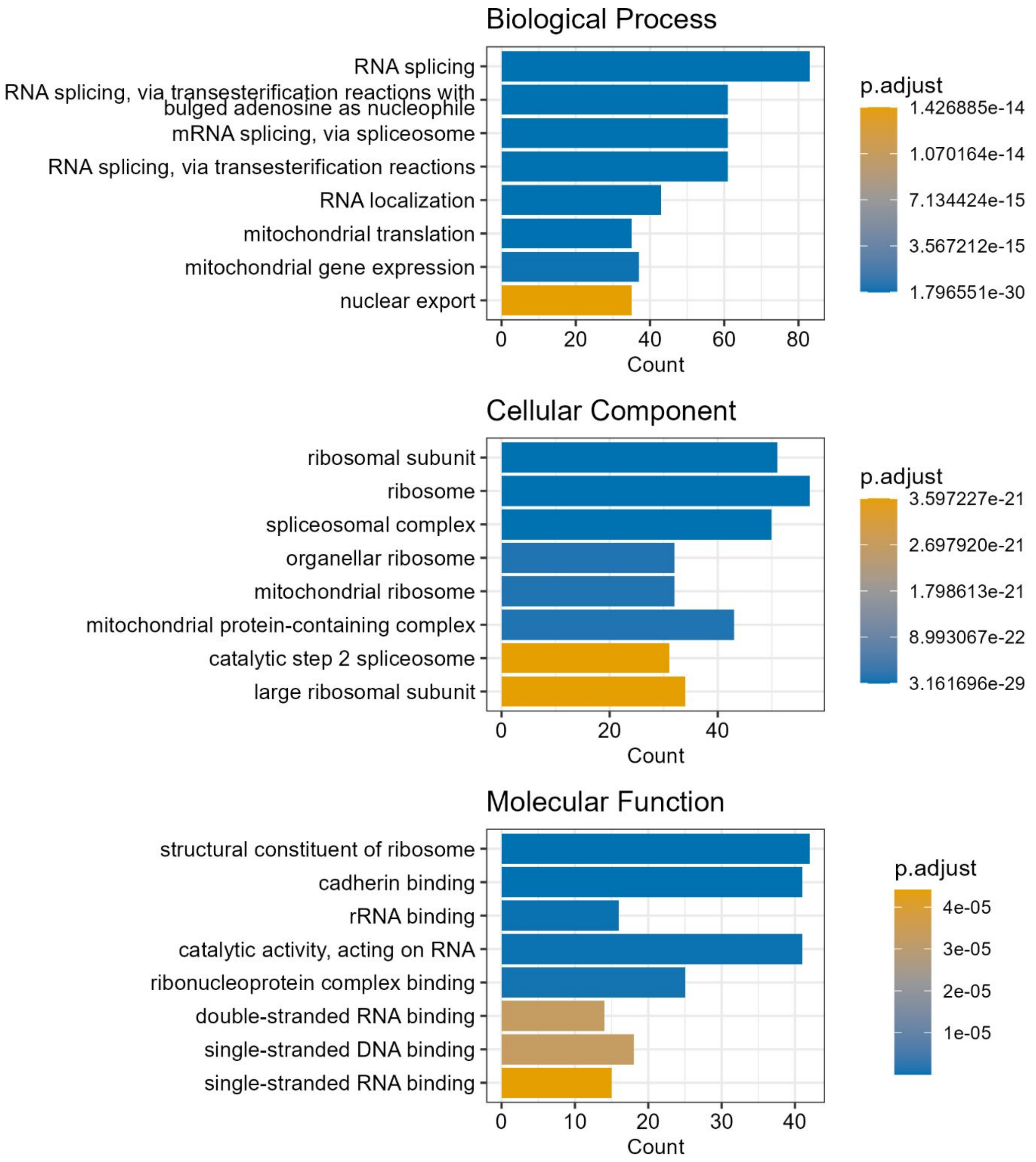

Down-regulated proteins

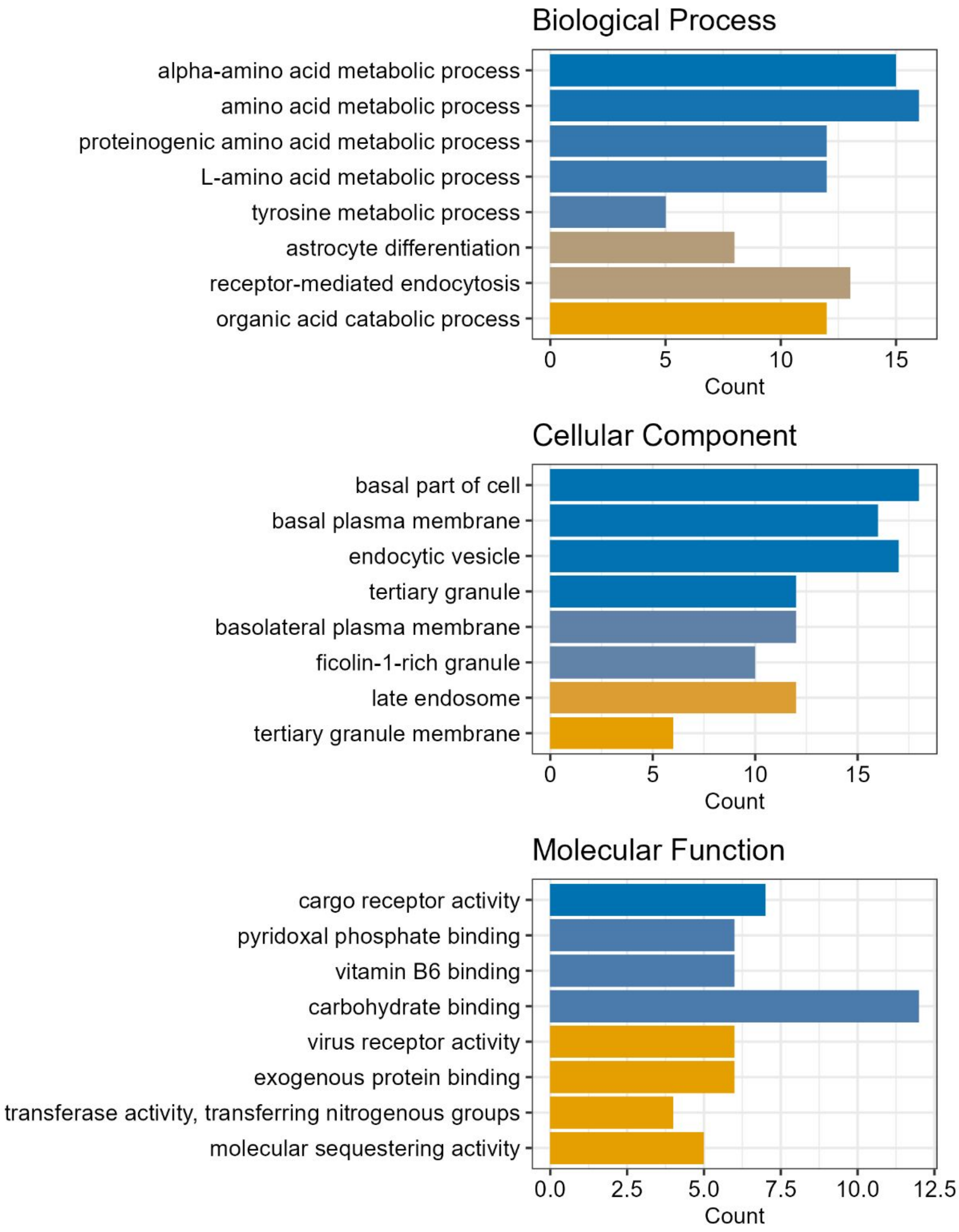

Figure S2. GO enrichment of significantly changed proteins between HCC and normal liver tissues.

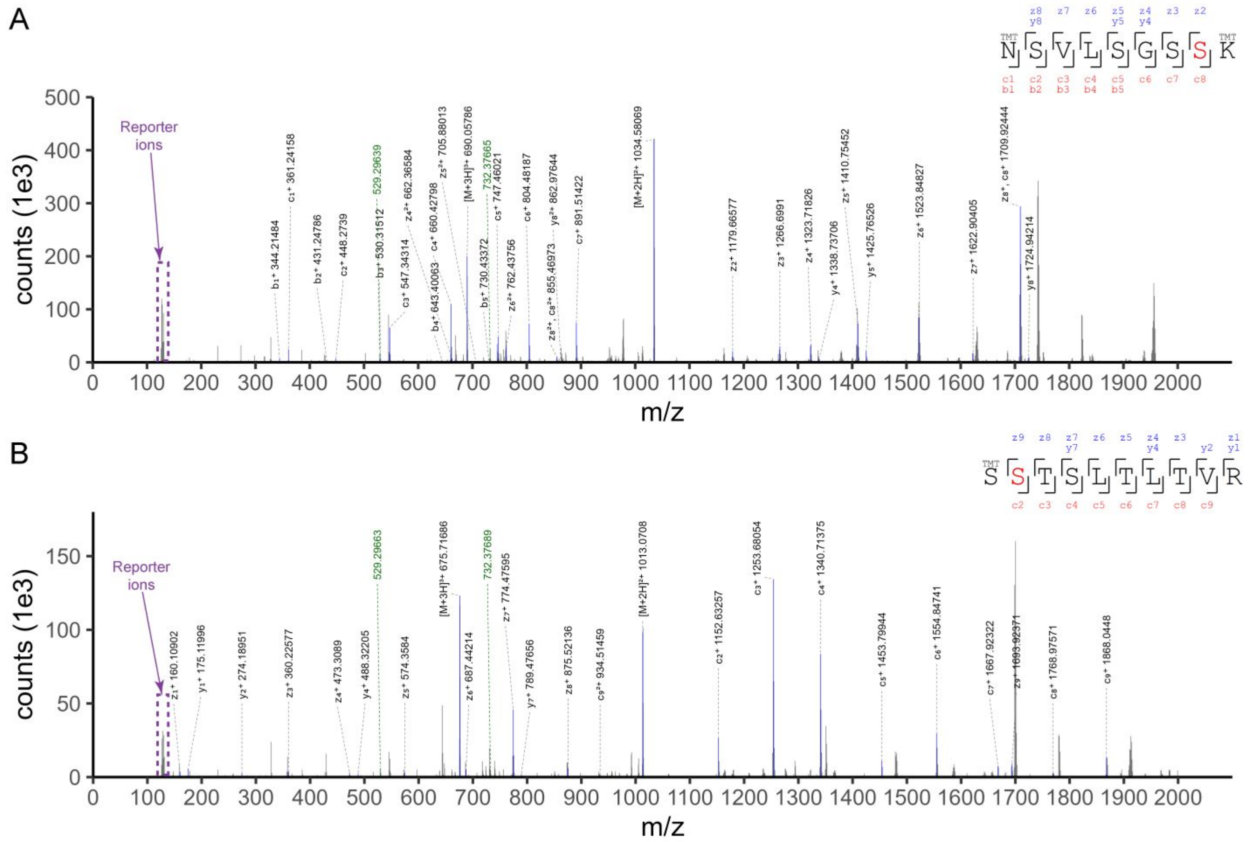

Figure S3. Representative mass spectra of two O-GlcNAcylated sites, i.e., S1550 (A) and S1864 (B) on Zinc finger protein 40 (HIVEP1). Matched b, y, c, and z ions are annotated, with O-GlcNAc site shown in red and peptide N-term TMT labeled. Two key fragments resulting from the PC-biotin-alkyne tag, i.e., m/z 732.37 and 529.29, are highlighted in green. TMT reporter ions are shown in purple.

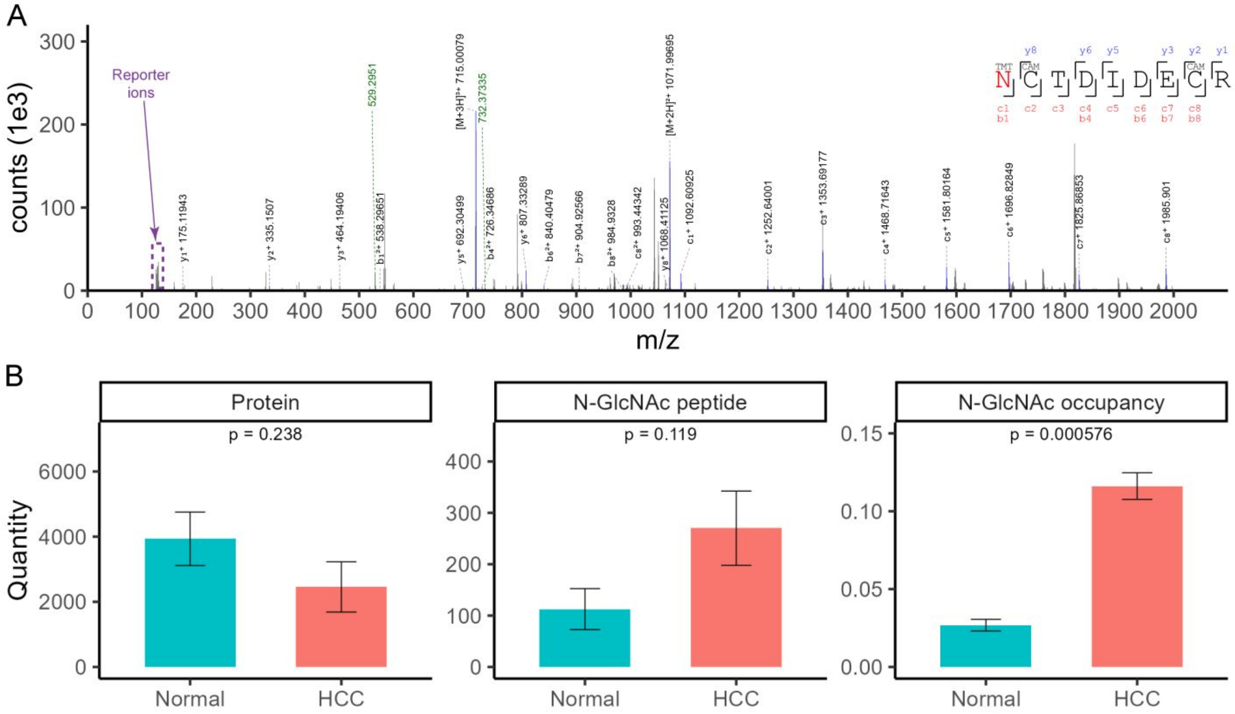

Figure S4. (A) Representative mass spectrum of GlcNAcylation on N1067 of Fibrillin-1 (FBN1) and (B) quantitation of the protein level, N1067 GlcNAcylation level, and GlcNAc site occupancy on N1067 of FBN1. Of note, the GlcNAcylated Asn in the peptide of FBN1 is highlighted in red in Figure S4A. Two Cys residues (i.e., C2 and C8) in the peptide are carbamidomethylated and the N-term is labeled by TMT. Two key fragments resulting from the PC-biotin-alkyne tag, i.e., m/z 732.37 and 529.29, are highlighted in green. TMT reporter ions are shown in purple.
